# Multi-Model Coupling Water Demand Prediction Optimization Method for Megacities Based on Time Series Decomposition

**DOI:** 10.1007/s11269-021-02927-y

**Published:** 2021-09-23

**Authors:** Xin Liu, Xuefeng Sang, Jiaxuan Chang, Yang Zheng

**Affiliations:** 1 School of Water Conservancy, North China University of Water Resources and Electric Power, Zhengzhou, 450046 China; 2 Research Office for Water Resources Management, China Institute of Water Resources and Hydropower Research, Beijing, 100038 China

**Keywords:** BLSTM, GRBFNN, Prediction interval, SARIMA, Water demand prediction, Wavelet transform

## Abstract

The water supply in megacities can be affected by the living habits and population mobility, so the fluctuation degree of daily water supply data is acute, which presents a great challenge to the water demand prediction. This is because that non-stationarity of daily data can have a large influence on the generalization ability of models. In this study, the Hodrick-Prescott (HP) and wavelet transform (WT) methods were used to carry out decomposition of daily data to solve the non-stationarity problem. The bidirectional long short term memory (BLSTM), seasonal autoregressive integrated moving average (SARIMA) and Gaussian radial basis function neural network (GRBFNN) were developed to carry out prediction of different subseries. The ensemble learning was introduced to improve the generalization ability of models, and prediction interval was generated based on student's t-test to cope with the variation of water supply laws. This study method was applied to the daily water demand prediction in Shenzhen and cross-validation was performed. The results show that WT is superior to HP decomposition method, but maximum decomposition level of WT should not be set too high, otherwise the trend characteristics of subseries will be weakened. Although the corona virus disease 2019 (COVID-19) outbreak caused a variation in water supply laws, this variation is still within the prediction interval. The WT and coupling models accurately predict water demand and provide the optimal mean square error (0.17%), Nash-Sutcliffe efficiency (97.21%), mean relative error (0.1), mean absolute error (3.32%), and correlation coefficient (0.99).

## Introduction

The water demand prediction in megacities has always been a hot topic for scholars. The population and buildings in megacities are dense, and the modernization degree is high. Therefore, the local water resources are scarce, and the water supply mainly depends on water diversion. For megacities, the water demand prediction is essential to improve the efficiency of water supply and make the water diversion plan of next year. Meanwhile, the water demand prediction is conducive to understand water supply and demand balance, so as to discover the shortage of water resources in advance.

In recent years, the data-driven models, such as regression (Vonk et al. 2019; Pesantez et al. 2020), artificial neural network (ANN) (Peng et al. 2020; Zubaidi et al. 2020; Salloom et al. 2021), time series (Xu et al. 2018; Tripathi et al. 2019; Smolak et al. 2020) and deep learning (Guo et al. 2018; Nasser et al. 2020; Du et al. 2021) models, have been widely used in water demand prediction. This is because the data-driven models will not be affected by the external physical environment. They can learn the potential correlation relationship between data to establish the quantitative relationship (Fu et al. 2019) between input and output, the modeling speed is fast and the prediction accuracy is high. Meanwhile, many good predicted results have been achieved in other fields, such as precipitation prediction (Wheeler et al. 2017), flood prediction (Khan et al. 2018) and water quality prediction (Ahmed et al. 2019).

However, as the units of time series get shorter, the generalization ability of data-driven models may deteriorate. According to a large number of experiments and studies, the non-stationarity of time series (Serinaldi et al. 2018; Wang et al. 2019) has a great influence on the prediction accuracy. The stationarity of annual data and monthly data is better than the daily data, so it is easier to construct the model using such data. For the daily water supply data in megacities, the stationarity of the data is poor. If the data-driven model is directly constructed using such data, the generalization ability of the model may deteriorate, which brings new challenges to the data-driven models. At the same time, the multi-collinearity (Bassiouni et al. 2016; Yoo and Cho 2019) among features can lead to the distortion of the multiple regression model. The ANN model is prone to overfitting (Baek and Kim 2018; Ghasemi et al. 2018) due to its global parameter adjustment, and the limited learning ability may result in local optimal solution. More importantly, the poor randomness and robustness of the models is also a key factor affecting the generalization ability of the models.

Although the stationarity of daily water supply data in megacities is not good, the data has the potential laws. If the appropriate method is used to decompose the time series, and the prediction and reconstruction of the subseries can improve the prediction accuracy of models. Hodrick-Prescott (HP) (Chen et al. 2020) decomposition method is commonly used at present, which decomposes time series into trend subseries (TS) and period subseries (PS). However, the experiment results show that the period laws of PS generated by HP method (HP-PS) are not good, and the significance level of the period laws can be affected by the irregular values. Sometimes, PS contains many irregular values, and the PS is very similar to Gaussian noise (Roberts et al. 2017). Therefore, in this study, wavelet transform (WT) (Rhif et al. 2019; Sakar et al. 2019) is used to decompose the time series into TS, PS and noise subseries (NS). The coupling model bidirectional long short term memory (BLSTM), seasonal autoregressive integrated moving average (SARIMA) and Gaussian radial basis function neural network (GRBFNN) are developed for the prediction of different subseries. The predicted results of WT and HP methods are compared, and the prediction interval is generated based on student's t-test (T-test). Through the decomposition of time series, and the prediction and reconstruction of subseries, the predicted values that have less error with measured values can be obtained. This research method can also provide reference for time series prediction in other fields.

## Material and Methods

### Study Area and Dataset

Shenzhen is located between longitudes of 113°43' and 114°38' E, and latitudes of 22°24' and 22°52' N, adjacent to Hong Kong. It is a sub-provincial city of Guangdong Province and the first city in China to be fully urbanized. Shenzhen is a port city with the largest number of ports, the largest number of entry-exit people and the largest traffic flow in China. Although the local water resources in Shenzhen are scarce and the water supply dispatching mainly depends on the water diversion, the rainfall in Shenzhen is very abundant. If water demand can be predicted accurately, the rainfall and water diversion can be used reasonably, and the water diversion plan of next year can be made scientifically. Therefore, the daily water demand prediction in Shenzhen is essential to improve the efficiency of water supply and provide support for water supply dispatching. Meanwhile, Shenzhen can also find out the potential water resources shortage in the future in advance, which is of great significance to the sustainable development of the society.

The data in this study are from the daily measured data without vacancy of Shenzhen Digital Water System from January 1, 2015 to December 31, 2020.

### Time Series Decomposition

#### Hodrick-Prescott (HP) Decomposition Method

There are two kinds of time series decomposition method: additive and multiplicative decomposition methods, and additive decomposition method is more commonly used, such as HP method (Eqs. 1 and 2). It is a widely used decomposition method, which decomposes time series into TS and PS. The two subseries are predicted respectively, and the final predicted values can be obtained by adding the predicted values of the two subseries. The TS is extracted first, and the PS is obtained by subtracting the TS from the original series.

1 $$\underset{ }{\mathrm{min}}\;\mathrm{Loss}=\mathrm{min}\left\{{\sum\nolimits_{t=1}^{n}}{\left({Y}_{t}-{T}_{t}\right)}^{2}+\lambda {\sum\nolimits_{t=3}^{n}}{\left[\left({T}_{t}-{T}_{t-1}\right)-\left({T}_{t-1}-{T}_{t-2}\right)\right]}^{2}\right\}$$

2 $$P=Y-T$$

where *Y*, *T* and *P* are the original series, the TS and the PS, respectively; *t* is the time, *n* is the length of the original series, and $\lambda$ is the smoothing factor. $\lambda$ is set to 800 in this study to preserve more the trend characteristic.

#### Wavelet Transform (WT) Decomposition Method

In general, the irregular values in time series are noise. In this study, the time series is decomposed into three subseries using Daubechies wavelet (Yelampalli et al. 2018) (Eq. 3) including TS, PS and NS. TS is obtained by filtering the wavelet coefficients through the soft threshold method. The vanishing moment, the maximum decomposition level (MDL) and the soft threshold is set to 22, 3 and 1, respectively. The high-frequency component is mostly noise. Therefore, after subtracting the TS from original time series, the non-common part of the high-frequency component is filtered to get the PS, and then the NS can be obtained (Eq. 4).

3 $${WT}_{f}\left(s,\tau \right)=\frac{1}{\sqrt{s}}{\int }_{-\infty }^{+\infty }f\left(t\right)\psi \left(\frac{t-\tau }{s}\right)dt$$

4 $$N=Y-T-P$$

where *WT*_*f*_, *s*, *t*, *τ* and *ψ* are the wavelet transform coefficient, the scale, time, deviation and wavelet base, respectively; *N* is the NS.

### The Coupling Model

#### Seasonal Autoregressive Integrated Moving Average (SARIMA)

Autoregressive integrated moving average (ARIMA) (Benvenuto et al. 2020; Nguyen 2020) model (Eq. 5) is a statistical machine learning model, which shows strong generalization ability in the prediction of time series with good stationarity. However, the ARIMA model has few parameters, including only autoregressive order, difference order and moving order. Therefore, SARIMA (Xu et al. 2019) model is developed in this study to carry out the prediction of PS. In addition to the three parameters of the ARIMA model, SARIMA model also has seasonal regression order, seasonal difference order and seasonal moving order, so this model has a strong learning ability to learn period laws. The maximum likelihood estimation (Eq. 6) is applied to solve model parameters. The standard for solving model parameters is Bayesian information criterion (BIC), and the candidate value intervals of parameter adjustment are [0, 10]. The difference order and seasonal difference order are set to 1 in this study.

5 $${{x}_{t}={\theta }_{1}{x}_{t-1}+\theta }_{2}{x}_{t-2}+\cdots +{\theta }_{p}{x}_{t-p}+{\mu }_{t}+{\alpha }_{1}{\mu }_{t-1}+{\alpha }_{2}{\mu }_{t-2}+{\alpha }_{q}{\mu }_{t-q}$$

6 $$L\left(\lambda \right)={\prod\nolimits_{i=1}^{n}}f\left({x}_{i}|\lambda \right) ,lnL\left(\lambda \right)={\prod\nolimits_{i=1}^{n}}lnf\left({x}_{i}|\lambda \right)$$

where *x*, *θ*, *μ* and *α* are the measured values at different time, the autoregressive coefficient, the noise at different time and the moving average coefficient, respectively; *L* and *λ* are likelihood function and parameters, respectively.

#### Bidirectional Ensemble Learning Long Short Term Memory (BELLSTM)

Long short term memory (LSTM) (Yu et al. 2019; Mu et al. 2020) model (Eqs. 9 and 10) is an improved recurrent neural network (RNN) model, so the generalization ability of LSTM is superior to that of RNN. The LSTM model can store memory that is constantly attenuated (Eqs. 7 and 8). The output of the model is not entirely dependent on the input of the current time, but is also affected by the output of the previous time and the attenuation memory. The weight of the recent memory is larger. Considering the non-linear problem of the time series, s*igmoid* and *tanh* (Eqs. 11 and 12) activation functions are developed to add the non-linear factors. To avoid the problem of abnormally large parameters passed between hidden layers, the hidden layer output normalization (Eq. 13) is applied to rectify the output so that the output is revised to [0, 1] before passing to the next layer. However, LSTM is a unidirectional model, and the unidirectional model cannot learn the bidirectional knowledge. In order to make the model learn bidirectionally (Chen et al. 2014; Zhang et al. 2018), the BLSTM model is developed to improve the learning ability of the model. The BLSTM model is constructed through 5 lag time, the model structure includes bidirectional LSTM with eight-layer network structure, one-layer feedforward neural network, and one-layer rectification neural network which is used to restore the dimensionality magnified by the bidirectional propagation.

7 $${\widetilde C}_t=Tanh\left(w_c\times\left[h_{t-1},x_t\right]+b_c\right)$$

8 $$C_t=f_{t-1}\times C_{t-1}+f_t\times{\widetilde C}_t$$

9 $${O}_{t}=Sigmoid\left({w}_{o}\times \left[{h}_{t-1},{x}_{t}\right]+{b}_{o}\right)$$

10 $${h}_{t}={O}_{t}\times Tanh\left({C}_{t}\right)$$

11 $$Sigmoid\left(x\right)=\frac{1}{1+{e}^{-x}}$$

12 $$Tanh\left(x\right)=\frac{{e}^{x}-{e}^{-x}}{{e}^{x}+{e}^{-x}}$$

13 $$norm=\frac{{{\varvec{Y}}}_{{\varvec{i}}}-{{\varvec{Y}}}_{min}}{{{\varvec{Y}}}_{max}-{{\varvec{Y}}}_{min}}$$

where *f*, *h*, *x*, *w*, *b*, ${\widetilde C}_t$, *C* and *O* are attenuation factor, the output of the hidden layer, the input at different time, the weight, the bias, the memory of the current time, attenuation memory and the output, respectively.

At present, data-driven models are usually single model or single coupling model. The limitation of this single model limits the improvement of model generalization ability. Therefore, the ensemble learning (EL) (Abbaszadeh et al. 2019; Alam et al. 2020) is introduced to improve generalization ability of model. During each round of training, the connections between some neurons are cut off at random with a certain probability, and the probability is set to 0.1 in this study. In this way, the trained model in each round is equivalent to a new model, and these new models are eventually integrated into the EL model. The model is solved by the least square (LS) method (Eq. 14). Meanwhile, genetic algorithm (GA) (Zhang et al. 2019) is developed to optimize the solution process of the model to solve the problem of local optimal solution.

14 $${minValue}_{LS}\left(\theta \right)=sum{\left|{\varvec{Y}}-{\varvec{P}}\right|}^{2}$$

where ***Y***, ***P*** and *θ* are measured value Tensor, predicted value Tensor and parameters, respectively.

#### Gaussian Radial Basis Function Ensemble Learning Neural Network (GRBFELNN)

Compared with ANN, radial basis function neural network (RBFNN) (Gholami et al. 2019) is a local parameter adjustment neural network, so the convergence speed of the model is faster and the performance is better. In this study, Gaussian (Xiang et al. 2020) function is applied as radial basis function to develop GRBFNN (Eqs. 15 and 16). The GRBFNN model is constructed through 5 lag time, the model structure includes RBFNN with three-layer network structure and two one-layer feedforward neural networks. Meanwhile, normalization, non-linear activation function, EL and GA are also introduced into the modeling process. The model is also solved by LS.

15 $$G\left(\mathrm{x}\right)=a{e}^{\frac{-{\left(x-b\right)}^{2}}{2{c}^{2}}}$$

16 $$f\left(x\right)={\sum\nolimits_{j=1}^{n}}{w}_{j}{G}_{j}\left(x\right)+B$$

where *G*, *c* and *B* are the Gauss function, the standard deviation and bias, respectively; *a* and *b* are constants.

### Model Evaluation Standard and Optimization

In this study, six standards are used to validate the performance of the models to avoid the contingency of a single evaluation standard. Mean square error (MSE), Nash-Sutcliffe efficiency (NSE), mean relative error (MRE), mean absolute error (MAE), Pearson correlation coefficient (*r*) and relative error (RE) (Eqs. 17-22) are used as the evaluation standards. They respectively represent the error, fitting degree, relative deviation degree, absolute deviation degree and correlation degree between measured and predicted values, and stability of model. Among them, RE between measured and predicted values is compared by violin plot. Considering the high time complexity of the element-by-element iterative calculation, deep learning Tensor is introduced as the data structure in this study. The one-dimensional Tensor is equivalent to the vector, and the multidimensional Tensor is equivalent to the matrix. All models and algorithms are developed by Python.

17 $$MSE=\frac{\sum {\left({\varvec{Y}}-{\varvec{P}}\right)}^{2}}{n}$$

18 $$NSE=\left\{1-\left[\frac{MSE\left({\varvec{Y}},{\varvec{P}}\right)}{Var\left({\varvec{Y}}\right)}\right]\right\}\times 100\mathrm{\%}$$

19 $$MRE=\frac{1}{n}{\sum\nolimits_{i=1}^{n}}\left|\frac{{{\varvec{Y}}}_{i}-{{\varvec{P}}}_{i}}{{{\varvec{Y}}}_{i}}\right|$$

20 $$MAE=\frac{1}{n}\sum \left|{\varvec{Y}}-{\varvec{P}}\right|$$

21 $$r=\frac{\sum \left({\varvec{P}}-\overline{{\varvec{P}} }\right)\left({\varvec{Y}}-\overline{{\varvec{Y}}}\right)}{\sqrt{\sum {\left({\varvec{Y}}-\overline{{\varvec{Y}} }\right)}^{2}\sum {\left({\varvec{P}}-\overline{{\varvec{P}} }\right)}^{2}}}$$

22 $$RE=\left|\frac{{\varvec{Y}}-{\varvec{P}}}{{\varvec{Y}}}\right|$$

where *var* and *n* are the variance and the length of data, respectively; ***P****i* and ***Y****i* are the value in time step *i*. $\overline{{\varvec{P}} }$ and $\overline{{\varvec{Y}}}$ are the average value of ***P*** and the average value of ***Y***, respectively.

## Results

### Time Series Decomposition

**Fig. 1:**
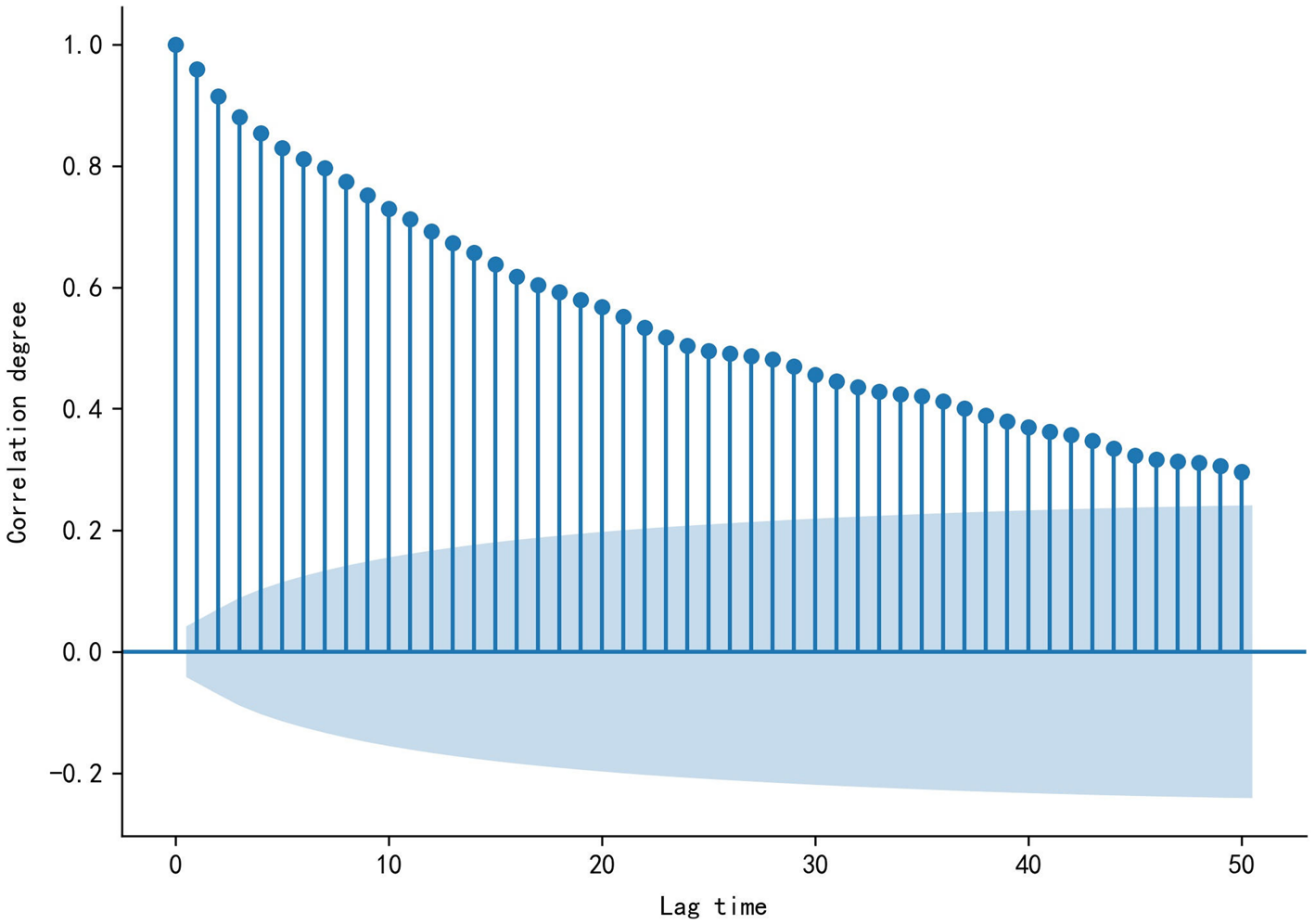
Correlation relationship plot of daily water supply data, the shadow region is the 95% confidence interval

Auto correlation function (ACF) (Dariane et al. 2018) (Eq. 23) is an effective method to understand stationarity of time series, which is helpful for mining the potential quantitative relationship of data. Figure 1 shows the autocorrelation relationship plot of data based on ACF.

23 $$ACF\left(k\right)=\frac{Cov\left({y}_{t} ,{y}_{t-k}\right)}{Var\left({y}_{t}\right)}$$

where *y*, *t*, *k*, *var* and *cov* are the measured value, the time, the number of lag time, the variance and the correlation coefficient.

As can be seen from Fig. 1, the daily water supply data in Shenzhen did not fall into the confidence interval after 50 lag time, indicating that the stationarity of this series was not good enough. Therefore, the time series needs to be decomposed, and the TS, PS and NS are respectively constructed the data-driven model by BELLSTM, SARIMA and GRBFELNN.

### Model Validation

The data in 2020 is taken as the testing set, and the data from January 1, 2015 to December 31, 2019 are divided into the training set and the validation set in a ratio of 8:2.

Table 1 shows the training results of the models, and all the models have good convergence. The accuracy of TS gives the better MSE, NSE, MRE, MAE and *r*. According to the five evaluation standards, the accuracy of WT-PS are obviously superior to that of HP-PS. The accuracy of WT-NS are not as good as that of WT-TS and WT-PS, because of the acute fluctuation of WT-NS. Table 2 shows the validation results of the models. The five evaluation standards of the WT-PS are optimal, and the five evaluation standards of HP-TS and WT-TS are very good. However, the accuracy of the HP-PS on validation set are significantly worse than that on training set, while the accuracy of the WT-NS on validation set are close to that on training set.

**Table 1:** The training results

Method	MSE/%	NSE/%	MRE	MAE/%	*r*
BELLSTM-HP-TS	0.04	98.70	0.03	1.48	0.99
SARIMA-HP-PS	0.94	20.58	0.15	7.14	0.61
BELLSTM-WT-TS	0.06	98.07	0.06	1.79	0.99
SARIMA-WT-PS	0.14	99.11	0.01	0.21	0.99
GRBFELNN-WT-NS	0.38	33.09	0.08	4.67	0.57

**Table 2:** The validation results

Method	MSE/%	NSE/%	MRE	MAE/%	*r*
BELLSTM-HP-TS	0.05	59.95	0.03	2.29	0.99
SARIMA-HP-PS	2.22	-121.21	0.27	13.09	-0.17
BELLSTM-WT-TS	0.06	80.52	0.03	2.23	0.97
SARIMA-WT-PS	0	100	0	0	1
GRBFELNN-WT-NS	0.28	35.69	0.06	3.64	0.59

**Fig. 2:**
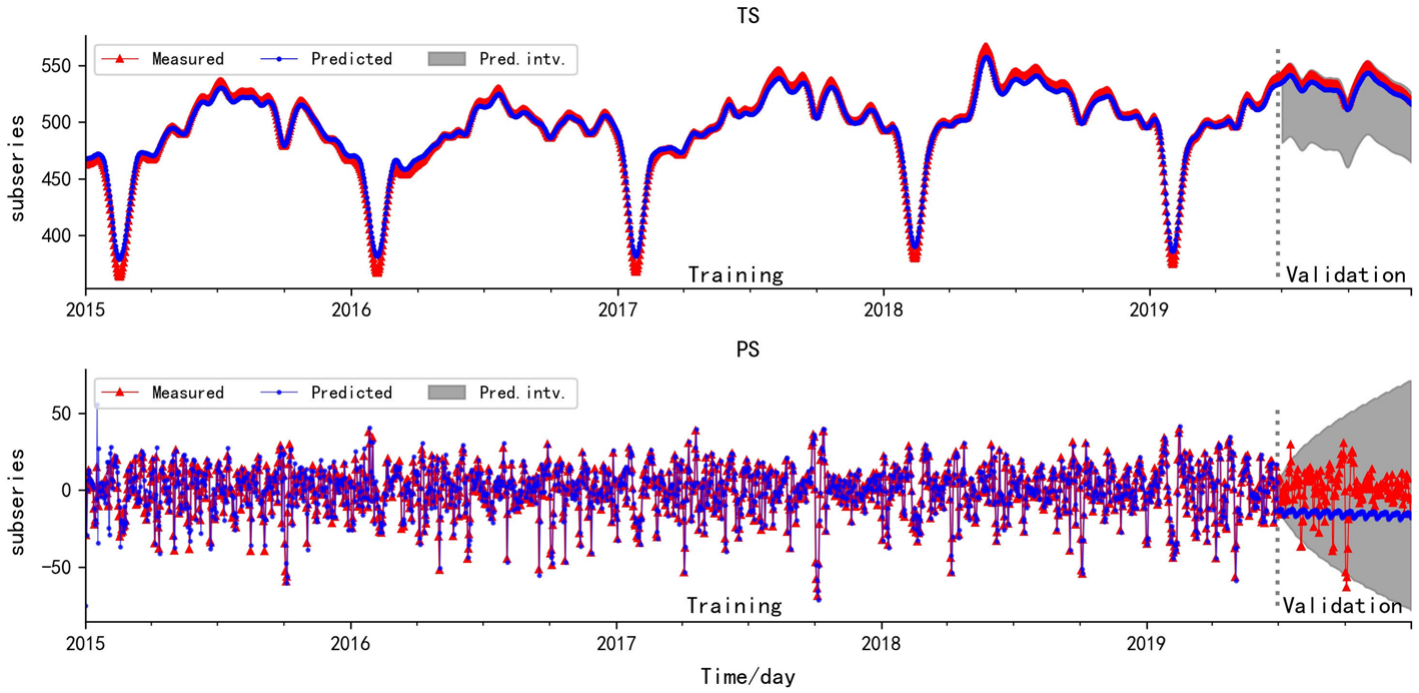
The fitting plot of HP-TS and HP-PS, the grey area is the prediction interval (Pred. intv.)

**Fig. 3:**
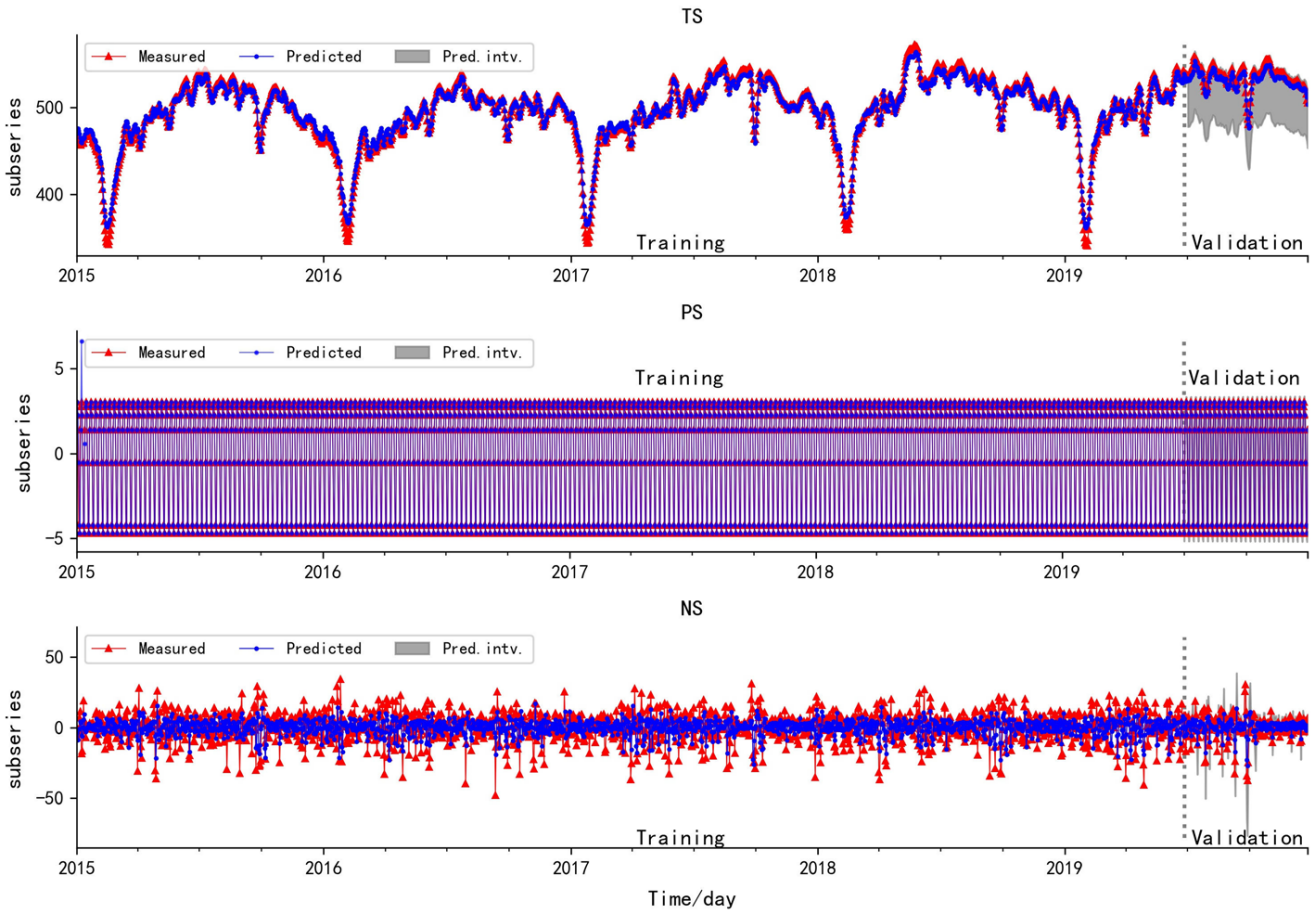
The fitting plot of WT-TS, WT-PS and WT-NS

According to Figs. 2 and 3, the validation results of HP-PS are seriously distorted. The MSE and MAE are large, and the NSE and *r* become negative, so validation results of HP-PS are completely unreliable. Nevertheless, the five evaluation standards of WT-PS are the best, which reveals that WT-PS is superior to HP-PS. This is because WT-PS has obvious period law, while HP-PS does not show an obvious period law, but shows acute fluctuation so that HP-PS is more like WT-NS. According to the evaluation standards of the HP-TS and WT-TS on validation set, the error and deviation degree between the predicted and the measured values are small, and the fitting degree and correlation degree are high. The accuracy of the BELLSTM model between the validation set and training set is close. These results indicate the BELLSTM model has strong learning ability. Similarly, the accuracy of the GRBFELNN model between the validation set and training set is close, which reveals that GRBFELNN model has a relatively strong learning ability for the time series with acute fluctuation.

In addition, the validation and training results of WT-PS show that SARIMA model has a strong learning ability for the time series with good stationarity, while the validation and training results of HP-PS reveal that SARIMA model shows poor performance for the prediction of time series with acute fluctuation.

### Prediction

Table 3 presents the predicted results of the subseries. According to the evaluation standards of the HP-TS and WT-TS, the error and deviation degree between the predicted and the measured values are small, and the fitting degree and correlation degree are high. The prediction accuracy of the BELLSTM model between the testing set and training set is close. Therefore, the BELLSTM model shows a very strong generalization ability, and the model is close to unbiased prediction. The MSE, NSE, MAE and *r* of the HP-TS and WT-TS are close, but the MRE of WT-TS is 0.79, which is 81% lower than that of HP-TS. The results indicate relative deviation degree of HP-TS between predicted and measured values is larger, which reveal that WT-TS is superior to HP-TS.

**Table 3:** The predicted results

Method	MSE/%	NSE/%	MRE	MAE/%	*r*
BELLSTM-HP-TS	0.14	97.93	4.16	2.74	0.99
SARIMA-HP-PS	1.46	-15.60	0.24	9.02	0.06
BELLSTM-WT-TS	0.17	97.39	0.79	2.98	0.99
SARIMA-WT-PS	0	100	0	0	1
GRBFELNN-WT-NS	0.60	25.00	0.09	5.10	0.50

Although the fluctuation degree of WT-NS is acute and the NSE and *r* of WT-NS are not good, the MSE, MRE and MAE are small. This reveals that the GRBFELNN has relatively strong generalization ability for the prediction of time series with acute fluctuation. However, the prediction accuracy of HP-PS is poor, so the SARIMA model shows poor generalization ability for the prediction of time series with acute fluctuation.

**Fig. 4:**
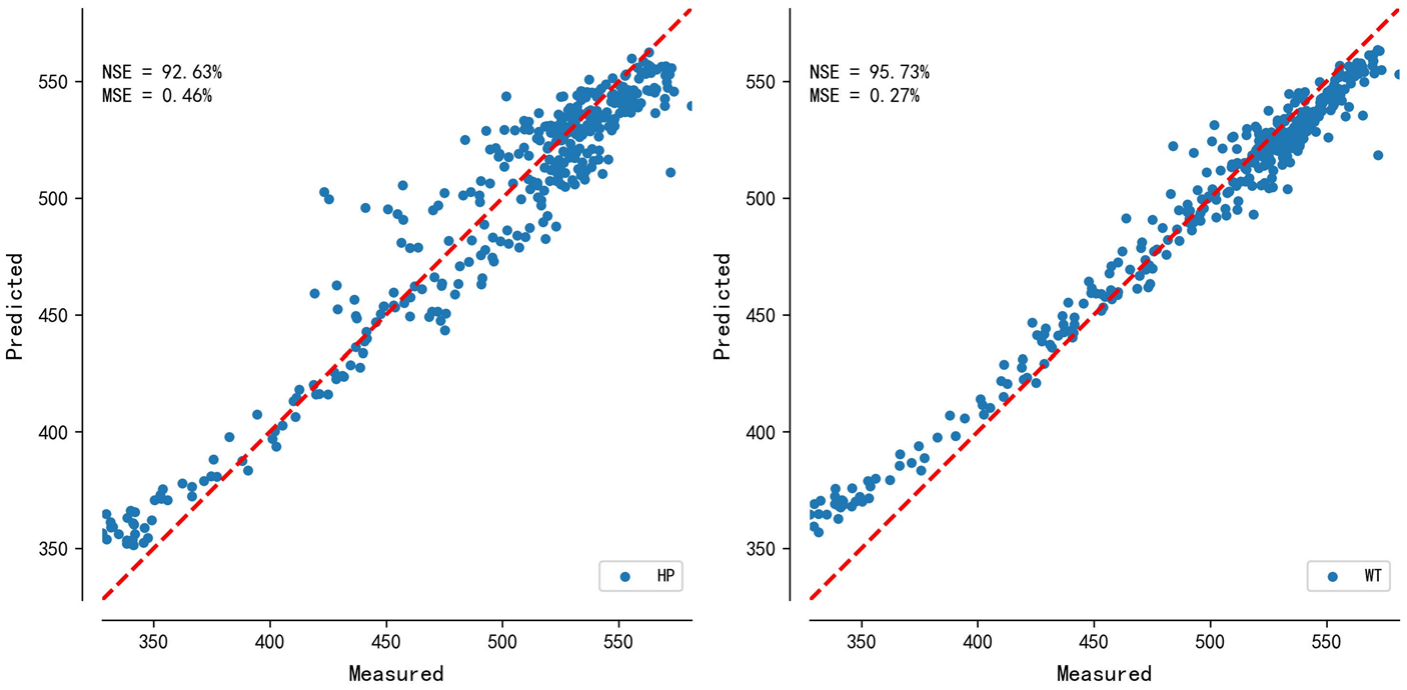
The distribution plot of predicted values and measured values

According to the evaluation standards of the subseries reconstruction, the predicted values of WT are superior to the predicted values of HP (Table 4). The predicted values of WT decomposition method have better error and fitting degree, which reveals that WT is superior to HP decomposition method. Figure 4 shows distribution plot of predicted values and measured values, the dots on red line indicate that the predicted values are equal to the measured values. The closer the dots are to the red line, the smaller the error between the predicted values and the measured values. Obviously, the dots of WT are superior to that of HP.

**Table 4:** Predicted results after subseries reconstruction and revision results based on T-test

Method	MSE/%	NSE/%	MRE	MAE/%	*r*
HP	0.46	92.63	0.29	5.25	0.96
HP-T	0.47	92.52	0.19	5.31	0.96
WT	0.27	95.73	0.33	4.01	0.99
WT-T	0.17	97.21	0.10	3.32	0.99

*HP-T* revision results of HP based on T-test, *WT-T* revision results of WT based on T-test

**Fig. 5:**
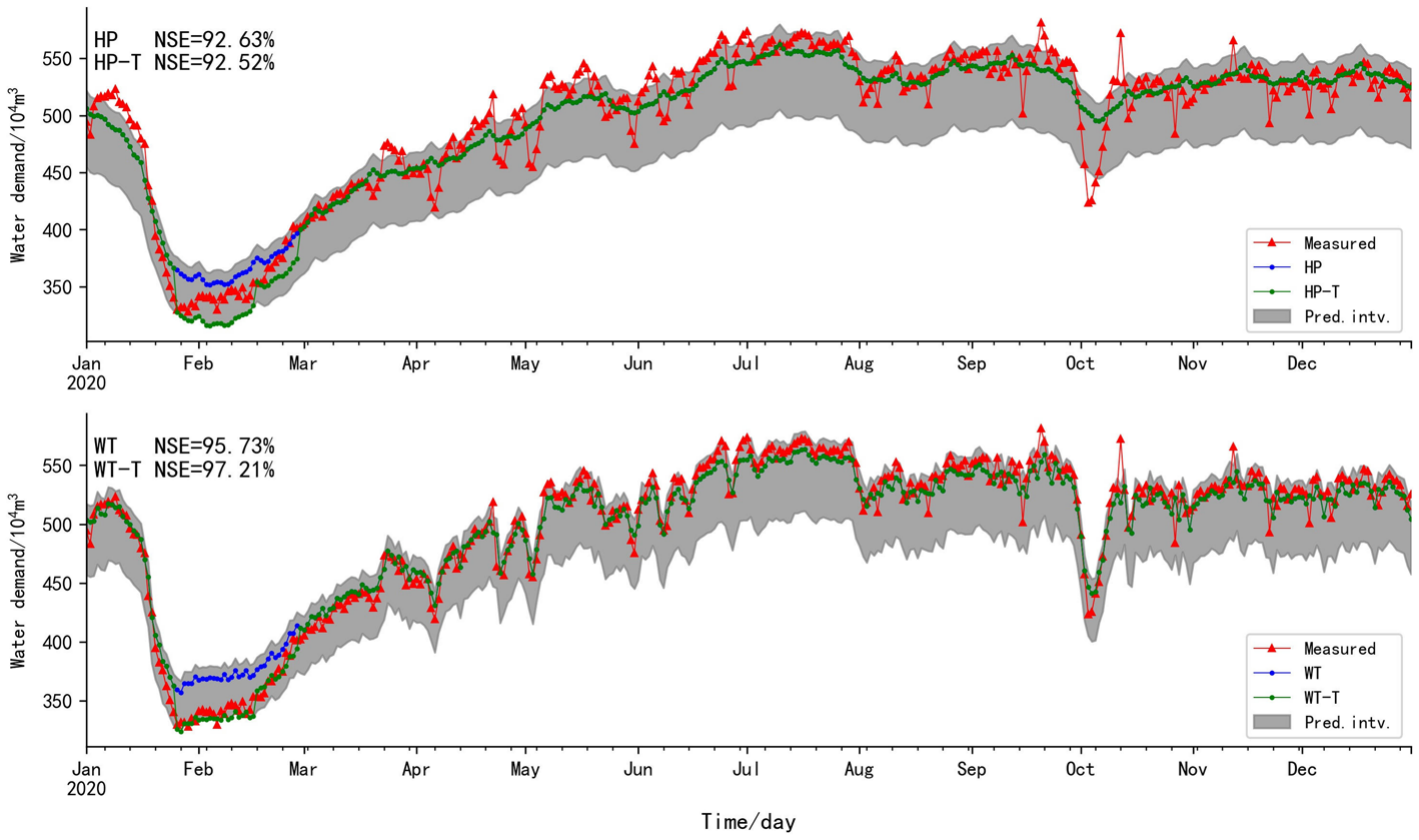
Fitting plot of predicted values and measured values, the blue line is the predicted values of the subseries reconstruction, the green line is revision results based on T-test

However, February 2020 was the period of corona virus disease 2019 (COVID-19) outbreak, the floating population could not return to Shenzhen. Shenzhen had only permanent residents, so the variation of water supply laws led to the deviation between the predicted values and the measured values (Fig. 5). Therefore, the prediction interval was estimated in this study based on the student's t-test (T-test) (Delacre et al. 2017) (Eq. 24).

24 $$t=\frac{\overline x-\mu}{s/\sqrt n}$$

where $\overline x$, $\mu$, *s* and *n* are the average value of the predicted data, average value of the measured data, standard deviation of the predicted data and length of predicted data, respectively.

According to the historical laws of water supply in Shenzhen, the water supply will gradually increase after the Spring Festival holiday and return to the normal level in the Lantern Festival. This variation reveals that although data-driven models can learn potential quantitative relationships between data, they cannot predict variation caused by emergency. Therefore, the prediction interval is estimated to cope with the variation of water supply laws, and it is used as the revision reference of the predicted data.

For HP, the laws of water supply are not well learned, for example, the decrease law of water supply during the National Day of China cannot been accurately predicted. Many measured values do not fall into the prediction interval. However, the fitting degree of predicted values of WT is better than that of HP, and most measured values fall into the prediction interval. Although the laws of water supply are affected by the emergency, the variation of water supply in February still conforms to the prediction interval (Fig. 5). When the country issued the quarantine policy during COVID-19, a large number of floating population could not return to Shenzhen. The decrease of water supply caused by the decrease of population in Shenzhen is predictable, so the lower envelope of the prediction interval should be used to revise the predicted values. The error between predicted values of WT-T and the measured values is small, and it gives the best MSE, NSE, MRE, MAE and *r* (0.17%, 97.21%, 0.1, 3.32% and 0.99, respectively) (Table 4). Therefore, the prediction interval is essential for water demand prediction. If the dispatching personnel can know the decrease degree of water demand, the amount of water diversion can be reduced. More water resources can be used in the summer peak to provide support for water supply dispatching. However, the error between the predicted values of HP-T and the measured values is still large, indicating that WT is superior to HP decomposition method.

**Fig. 6:**
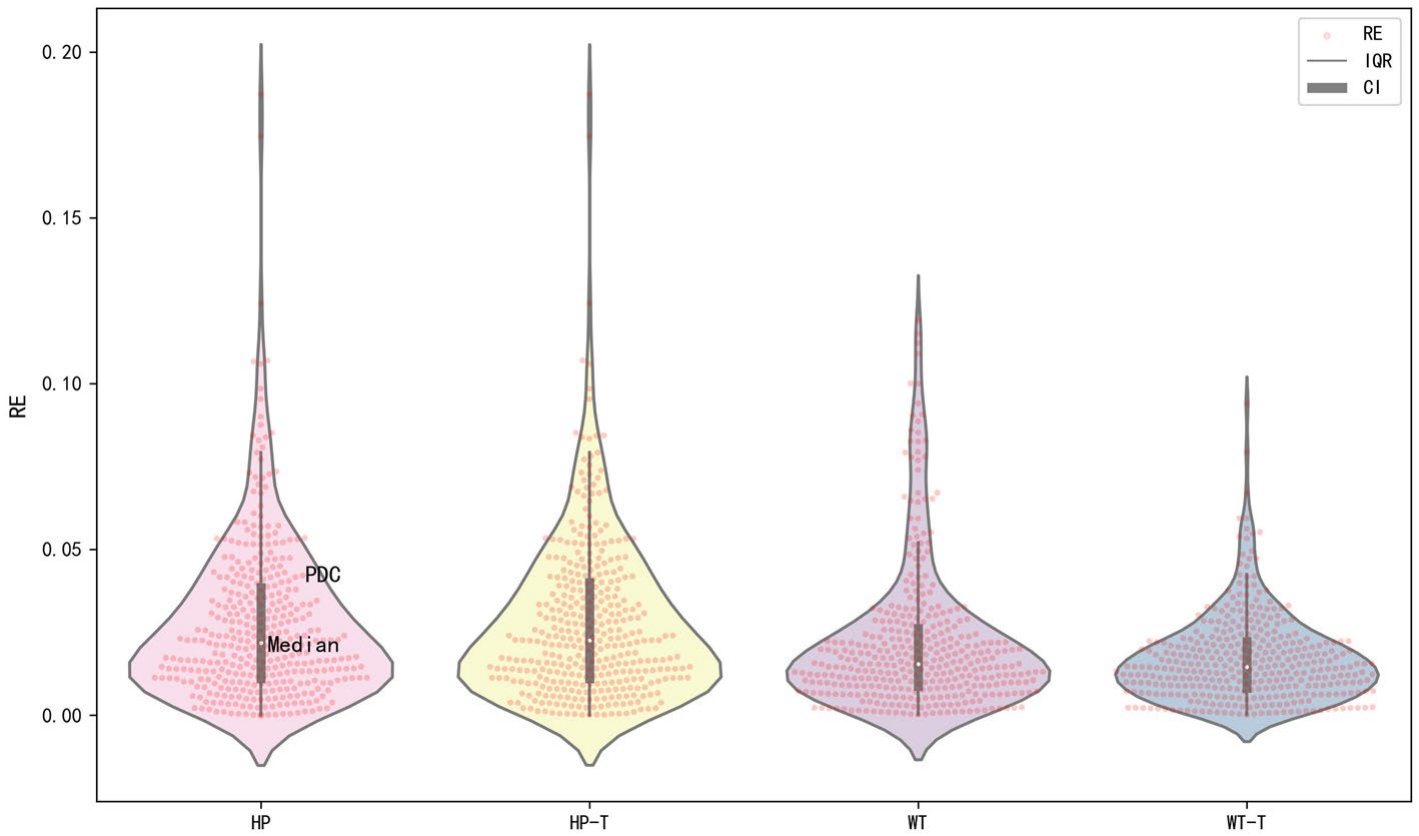
The violin plots of different methods, the outer curve is the probability density curve (PDC), the red dots are RE data, the thin line is the 95% confidence interval, the thick line is IQR, the white dot is the median

The stability of the methods is validated by the RE between each predicted value and measured value. The violin plots of different methods are shown to compare the RE distributions (Fig. 6), and Table 5 shows the violin parameters. Compared with the violin parameters of HP, although the confidence interval lower limit (CILL) of WT and HP are both 0, the violin parameters of WT have smaller confidence interval upper limit (CIUL), upper quartile (UQ), median and lower quartile (LQ). The confidence interval (CI) and the interquartile range (IQR) of WT are smaller than that of HP. These results reveal that the RE values of WT are smaller and RE distribution of WT is denser, so the stability of WT is superior to that of HP decomposition method. Compared with the violin parameters of WT, the violin parameters of WT-T have smaller CIUL, UQ, median and LQ. The CI and the IQR of WT-T are smaller than that of WT. These results reveal that the violin parameters of WT-T is superior to that of WT and RE distribution of WT-T is denser. The violin parameters of HP-T are inferior to that of HP, while the violin parameters of WT-T are superior to that of WT. The WT-T gives the best CIUL, UQ, median, LQ and CILL (4.34%, 2.23%, 1.46%, 0.82% and 0, respectively). The violin plots of HP and HP-T are almost the same, while the violin plot of WT-T is significantly superior to that of WT.

**Table 5:** The violin parameters

Method	CIUL/%	UQ/%	Median/%	LQ/%	CILL
HP	7.97	3.85	2.19	1.10	0
HP-T	8.33	3.99	2.25	1.10	0
WT	5.22	2.62	1.55	0.88	0
WT-T	4.34	2.23	1.46	0.82	0

*CI* CIUL-CILL, *IQR* UQ-LQ

### Cross-validation

In order to further validate the effectiveness of the methods, the entire dataset is divided into training set and testing set according to 7:3 for cross-validation.

Table 6 shows the prediction results of cross-validation, the prediction accuracy of cross-validation is similar to that of Table 3. The predicted accuracy of WT-PS is superior to that of HP-PS. Although the predicted results of the HP-TS and WT-PS are good, the MRE of WT-TS decreases by 80.43% compared with the MRE of HP-TS, which shows that the relative deviation degree between the predicted values of WT-TS and the measured values is smaller. Because the period laws of HP-PS are not obvious, the predicted accuracy of SARIMA model is poor. However, the period laws of WT-PS are obvious, so the predicted accuracy of SARIMA model is good. According to the prediction accuracy of WT-NS, the error and deviation degree between the predicted values and the measured values are small, which shows that GRBFELNN model has relatively strong generalization ability for the prediction of time series with acute fluctuation.

**Table 6:** The cross-validation results

Method	MSE/%	NSE/%	MRE	MAE/%	*r*
BELLSTM -HP-TS	0.10	97.78	2.35	2.37	0.99
SARIMA-HP-PS	1.42	-14.02	0.24	8.45	0.01
BELLSTM -WT-TS	0.12	97.25	0.46	2.55	0.99
SARIMA-WT-PS	0	100	0	0	1
GRBFELNN-WT-NS	0.49	30.97	0.08	4.76	0.55

**Fig. 7:**
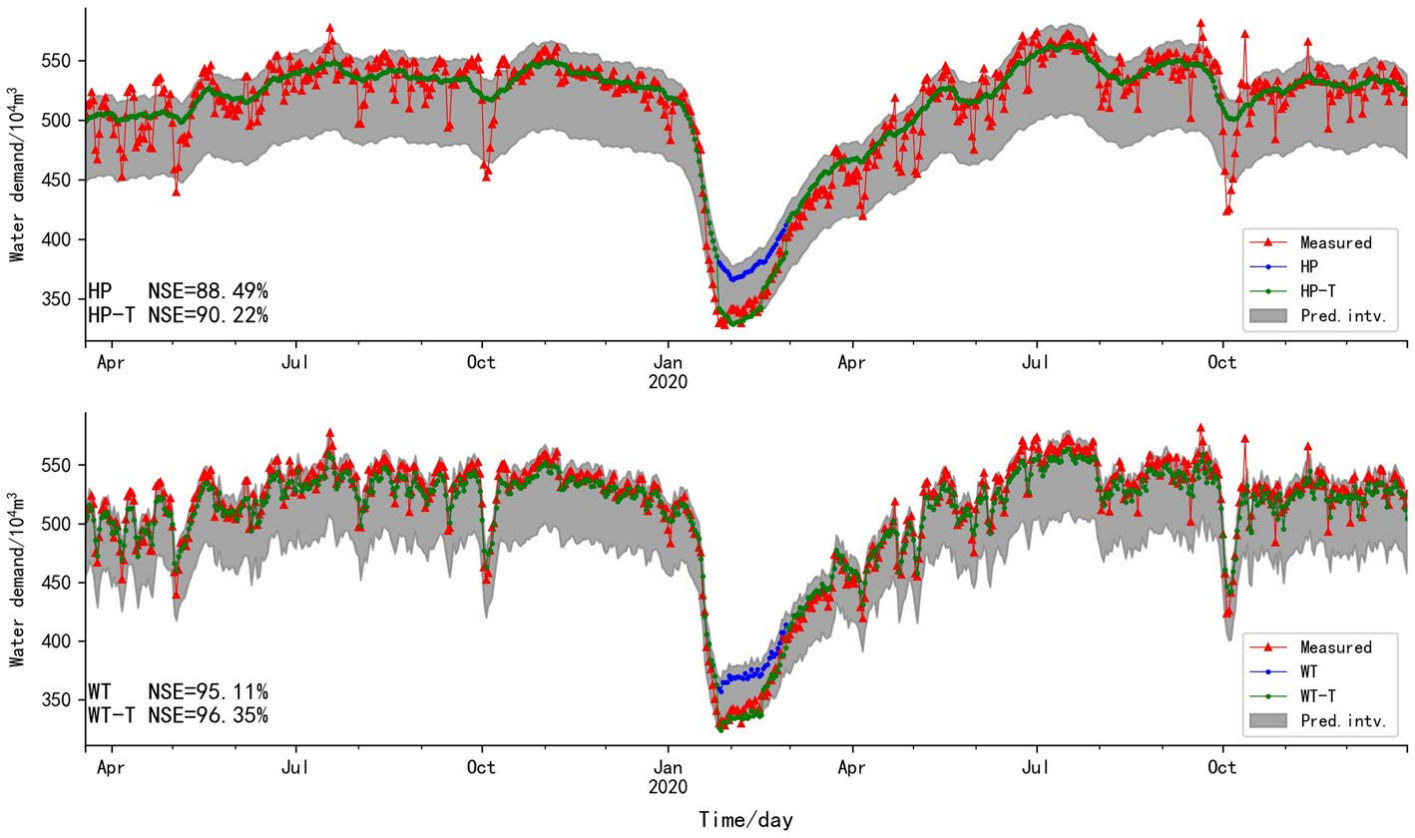
The fitting plot of predicted values after subseries reconstruction and measured values in cross-validation

The fitting degree of HP is still not as good as that of WT (Fig. 7). For HP, the statutory holiday, such as May Day and National Day of China, the decrease laws of water supply cannot be accurately predicted. Nevertheless, the prediction interval of HP in cross-validation is better than prediction interval in Fig. 5, which shows that the prediction interval of HP is affected by data length. For WT, the fitting degree between predicted values and measured values is high, and the decrease laws of water supply in statutory holiday can be accurately predicted. The prediction interval of WT is still good, indicating it is not affected by data length, so WT is more reliable than HP decomposition method. Meanwhile, the fitting degree of WT-T is higher than that of WT. Therefore, WT is superior to HP decomposition method, and the prediction interval helps to revise the prediction error caused by the variation of the water supply laws. The predicted results after subseries reconstruction and revision results based on T-test are showed in Table 7.

**Table 7:** Predicted results after subseries reconstruction and revision results based on T-test in cross-validation

Method	MSE/%	NSE/%	MRE	MAE/%	*r*
HP	0.48	88.49	0.27	5.05	0.95
HP-T	0.41	90.22	0.11	4.59	0.95
WT	0.21	95.11	0.20	3.55	0.98
WT-T	0.15	96.35	0.07	3.16	0.98

**Fig. 8:**
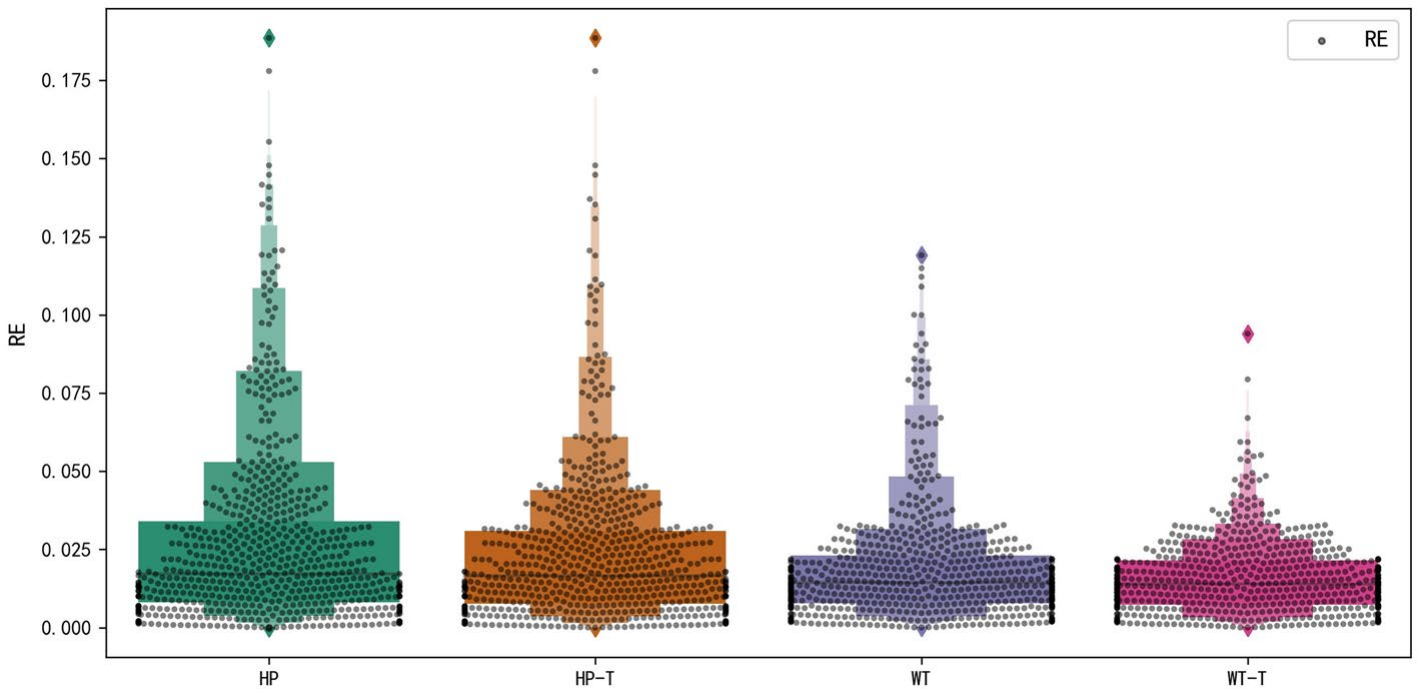
The box plots of different methods

Figure 8 shows box plots of the different methods, and the box height in the shadow region represents the density of RE data. The smaller the box height, the larger the density of RE data. The RE maximum value of HP and HP-T is very close, and the RE distribution is almost same. The box plot of HP-T is superior to that of HP only in the interval [0.03, 0.06]. This is because the NSE between predicted values and measured values of HP is not high enough. However, the box plot of WT is obviously superior to that of HP. The RE maximum value of WT is smaller and the density of RE is larger. Compared with box plot of WT, the box height of WT-T decreases obviously, the maximum value of RE decreases obviously, and the RE distribution is denser. Most dots distributed in the interval [0, 0.03]. In conclusion, WT is superior to HP decomposition method.

## Discussion

According to the above analysis, the error of HP is larger mainly because of the poor prediction accuracy of HP-PS. However, the above results have shown that the GRBFELNN model has a strong generalization ability for WT-NS. Therefore, the GRBFELNN model is used to carry out the prediction of HP-PS to compare the generalization ability of GRBFELNN and SARIMA models.

Table 8 presents the prediction results after subseries reconstruction. The five evaluation standards of BELLSTM-GRBFELNN-T-test (B-G-T) are superior to that of BELLSTM-SARIMA-T-test (B-S-T), which shows that GRBFELNN model has better generalization ability than SARIMA model for the prediction of time series with acute fluctuation. However, compared with the predicted accuracy of WT-T (Table 4), the prediction accuracy of B-G-T is inferior to that of WT-T, indicating that WT is superior to HP decomposition method.

**Table 8:** Predicted results after subseries reconstruction of different methods

Method	MSE/%	NSE/%	MRE	MAE/%	*r*
B-S-T	0.47	92.52	0.19	5.31	0.96
B-G-T	0.28	95.53	0.11	4.12	0.98

In addition, during the time series decomposition, it is found that the WT-PS is relatively stable for different MDL, but WT-TS is more sensitive to different MDL. The different MDL has a large influence on the trend characteristics of WT-TS. In this study, the MDL is set to 5 to decompose time series again (WT2) to compare the prediction accuracy with the original MDL (WT1).

**Fig. 9:**
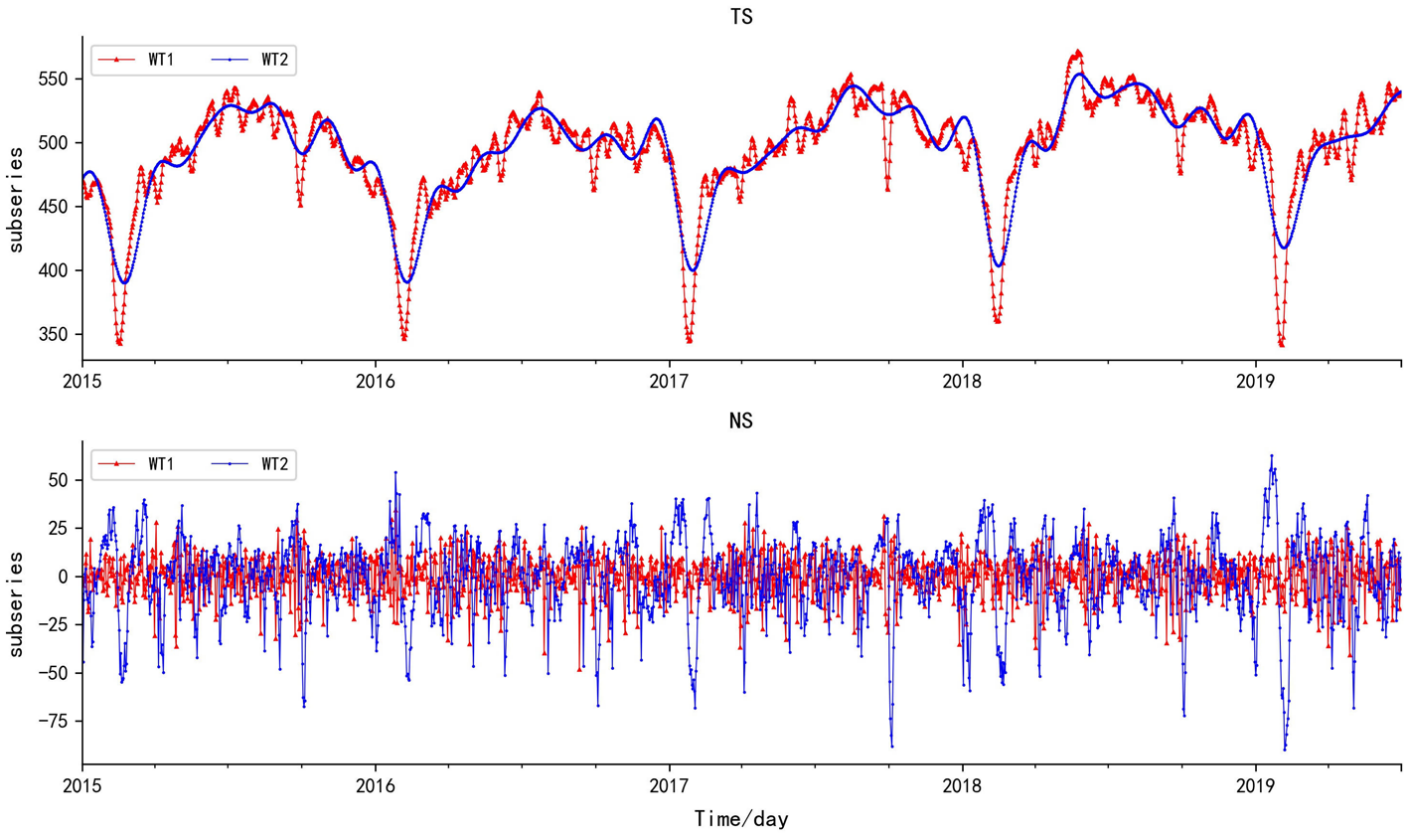
The comparison of TS and NS of WT1 and WT2

**Fig. 10:**
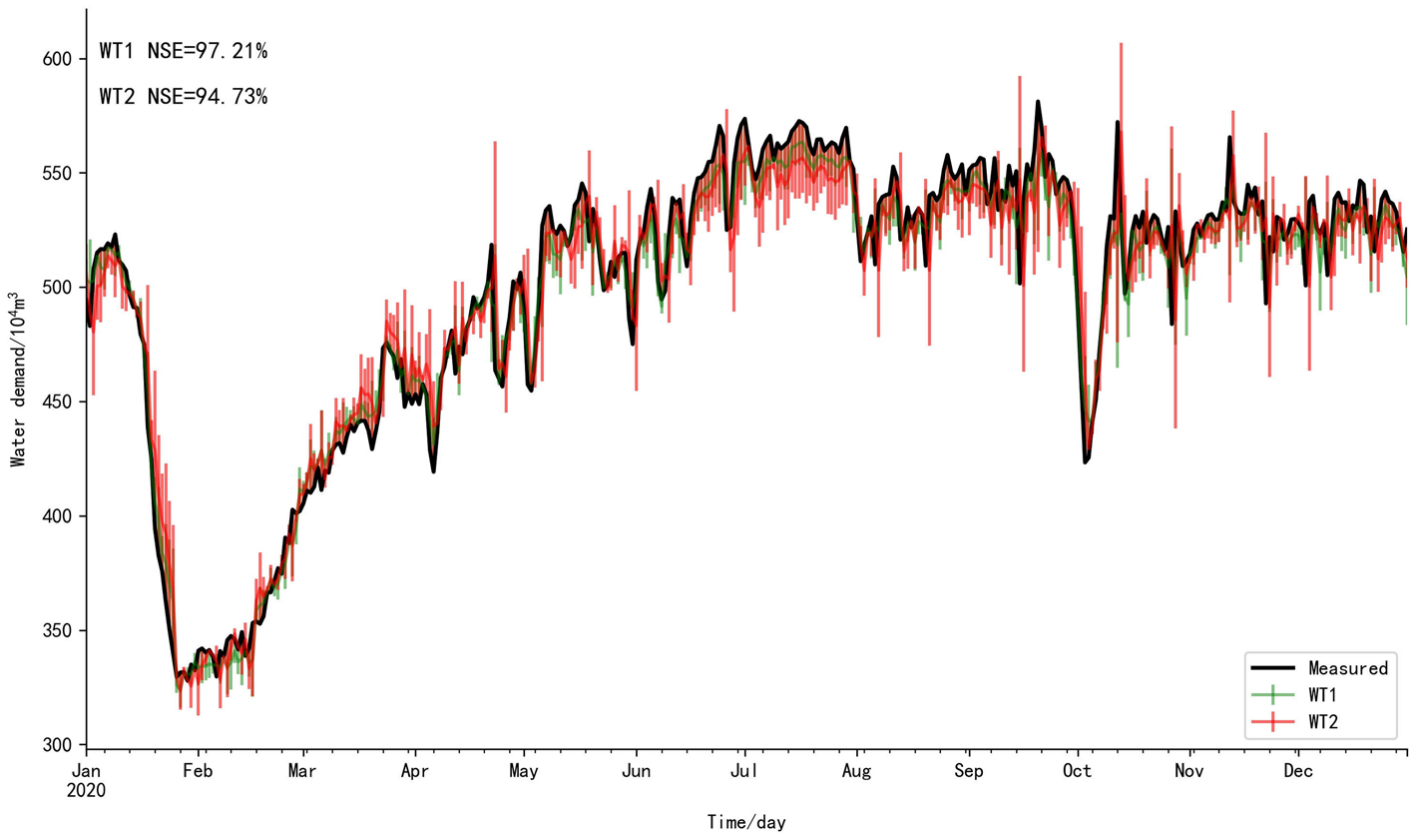
The absolute error bar plot of predicted results of WT1 and WT2

Table 9 presents the predicted results of different MDL. The prediction accuracy of WT1-T is superior to that of WT2-T. Although the stationarity of the WT2-TS is better, the stationarity of the WT2-NS significantly decreased (Fig. 9). The WT2-TS is too smooth, and the trend characteristics are weakened to a certain extent. The fluctuation degree of WT2-NS is more acute, the maximum and minimum values of WT2-NS are stretched along the vertical axis. Since the predicted accuracy of WT1-T and WT2-T are not much different, the absolute error bar plot is applied to compare their predicted results (Fig. 10).

**Table 9:** Predicted results after subseries reconstruction of WT1-T and WT2-T

Method	MSE/%	NSE/%	MRE	MAE/%	*r*
WT1-T	0.17	97.21	0.1	3.32	0.99
WT2-T	0.33	94.73	0.12	4.46	0.97

*WT1-T* revision results of WT1 based on T-test, *WT2-T* revision results of WT2 based on T-test

Compared with error bars of WT1, the error bars of WT2 become longer, and the error bars increase obviously at some moments. Therefore, WT should not set MDL too high. Based on repeated experiments, it is found that the optimal MDL is 3, so as to ensure the trend characteristics of the TS and reduce the fluctuation of the NS.

Finally, the BELLSTM and GRBFELNN models are used to carry out the water demand prediction without time series decomposition. The results are showed in Table 10. Prediction accuracy of WT-T is superior to that of BELLSTM-T and GRBFELNN-T. Therefore, the prediction method with time series decomposition is superior to that without time series decomposition. Meanwhile, prediction accuracy of BELLSTM-T is superior to that of GRBFELNN-T, indicating the generalization ability of data-driven model with the ability of bidirectional propagation is better.

**Table 10:** Predicted results of three methods

Method	MSE/%	NSE/%	MRE	MAE/%	*r*
WT-T	0.17	97.21	0.10	3.32	0.99
BELLSTM-T	0.39	93.34	0.22	5.01	0.97
GRBFELNN-T	0.55	90.55	0.27	6.28	0.96

## Conclusions

In this study, ACF is used to understand the stationarity of time series to provide reference for the construction of data-driven models. Based on the results of ACF, the time series decomposition methods based on HP and WT are proposed to solve the non-stationarity problem of time series, so as to improve the prediction accuracy of data-driven models. The BELLSTM, SARIMA and GRBFELNN models are developed for prediction of TS, PS and NS, respectively. Non-linear activation functions are introduced to increase the non-linear factors, and normalization between hidden layers is used to rectify the output. At the same time, EL is introduced to the modeling process to improve the generalization ability of the models, and GA is developed to optimize the solution process of the models. The prediction interval is generated based on T-test to cope with the variation of water supply laws. The methods are applied to daily water demand prediction in Shenzhen and cross-validation is performed. The five evaluation standards are used and multiple statistical figures, such as violin plot, box plot and absolute error bar plot, are drawn to clearly display and compare prediction accuracy of different models.

The results show that WT is superior to HP decomposition method. Although there is a variation in the water supply laws, its distribution still conforms to the prediction interval, which reveals that prediction interval is essential to revise the prediction error caused by the variation of the water supply laws. WT-T gives the best MSE, NSE, MRE, MAE and *r* (0.17%, 97.21%, 0.1, 3.32% and 0.99, respectively). The fitting degree and correlation degree between the predicted values and the measured values are the highest, and the error and deviation degree are the least. The prediction results are close to the unbiased prediction. According to violin plot, the RE distribution of WT-T is best, and WT-T gives the best CIUL, UQ, median, LQ and CILL (4.34%, 2.23%, 1.46%, 0.82% and 0, respectively). In cross-validation, the predicted results of WT-T are still the best, and the prediction interval of WT is superior to that of HP, indicating that WT is more reliable than HP.

For the results of the subseries, BELLSTM model presents the strong generalization ability for the prediction of TS. SARIMA model has strong generalization ability for WT-PS, but poor generalization ability for HP-PS. GRBFELNN model shows strong generalization ability for the prediction of WT-NS, and prediction accuracy of B-G-T is superior to that of B-S-T. These results reveal that GRBFELNN model has a strong generalization ability for the prediction of time series with acute fluctuation, and SARIMA model is more suitable for the prediction of time series with obvious period laws. MDL has less effect on the PS, but larger effect on the TS. By comparing the influence of MDL on the prediction accuracy, the best value of MDL is 3. Too high MDL will weaken the trend characteristics of TS, aggravate the fluctuation degree of NS, and the prediction accuracy will decrease.

## Data Availability

Custom code written in Python 3 was developed for this study.
